# Utility of the Lactate/Albumin Ratio as a Predictor for Mortality in Necrotizing Fasciitis Patients

**DOI:** 10.1155/2021/3530298

**Published:** 2021-10-13

**Authors:** Kiew-Kii Lau, Cheng-Ting Hsiao, Wen-Chih Fann, Chia-Peng Chang

**Affiliations:** ^1^Department of Emergency Medicine, Chang Gung Memorial Hospital, Chiayi, Taiwan; ^2^Department of Medicine, Chang Gung University, Taoyuan, Taiwan

## Abstract

**Background:**

The lactate/albumin (L/A) ratio has been proposed as a prognostic marker because the ratio is associated with multiple organ failure and mortality in critically ill patients. We aimed to investigate the clinical utility of the L/A ratio as a good prognostic indicator of mortality in a cohort of necrotizing fasciitis patients.

**Method:**

This retrospective study was conducted in two tertiary hospitals in Taiwan between 2015 and 2020. We reviewed adult patients with measured serum lactate and albumin on the emergency department (ED) arrival to evaluate the prognostic performance of the lactate and lactate/albumin (L/A) ratio for outcome prediction.

**Result:**

Of the 262 NF patients, 20 (7.63%) died in the hospital. The area under the receiver operating characteristic curve (AUROC) value of the L/A ratio (0.76, 95% confidence interval [CI] 0.69–0.81, *P* < 0.01) was higher than lactate alone (0.71, 95% CI 0.65–0.74 *P* < 0.01) for predicting in-hospital mortality. The optimal cutoff of the L/A ratio was 1.61. The AUROC value of the L/A ratio was better than lactate alone regardless of normal lactate level. The cutoff of L/A ratio and hypoalbuminemia showed further discriminative value for in-hospital mortality even in patients with normal lactate levels.

**Conclusion:**

The prognostic performance of the L/A ratio was superior to a single measurement of lactate for predicting in-hospital mortality and intensive care unit (ICU) lengths in necrotizing fasciitis patients. Aggressive intervention and intensive care were necessary for high-risk NF patients upon ED arrival.

## 1. Introduction 

Necrotizing fasciitis (NF) is a rare, rapidly progressive infection of subcutaneous tissue and fascia, characterized by widespread fascial necrosis [1]. The treatment of choice for NF is rapid surgical debridement and broad-spectrum antibiotic therapy [2]. Even under rapid and timely management, the risk of mortality and morbidity, such as amputation and multiorgan dysfunction, remains high [3–5]. Therefore, predictive biomarkers of mortality in NF patients are important for timely management.

Among widely used laboratory markers, band polymorphonuclear neutrophils >10%, serum creatinine level >2 mg/dL, hyperlactatemia, Laboratory Risk Indicator of Necrotizing Fasciitis (LRINEC) score >8, and serum albumin levels have been identified in association with mortality [6–8]. Elevated lactate was shown to be independently associated with mortality rate in critical ill patients [9]. Even intermediate initial serum lactate was an indicator of mortality of organ dysfunction and shock in severe sepsis patients in the emergency department (ED) [10]. In hospitalized patients, increased lactate indicated high mortality, mechanical ventilation, vasopressor requirement, and a high incidence of ICU admission [11–13]. Besides, serum albumin levels may also serve as a prognostic factor for critical patients, and the diagnostic performance of this parameter in critical care is well known [14–16]. The clinical significance of serum lactate and albumin was established over the last decade. Trends in lactate concentration over time reflect the clinical response of patients to resuscitation and surgical intervention. However, lactate and albumin level is not routinely monitored at the time of patient arrival as standard of care. Emergency department (ED) physicians are concerned with the resuscitation and identification risk of NF patients. Early recognition of NF patients who are at high risk for mortality allows for timely changes in therapy and improves the overall outcomes. We try to measure the association of the initial serum lactate and albumin level in the ED with in-hospital mortality of NF. Our objective in this study was to retrospectively evaluate whether the lactate/albumin ratio in ED is associated with increased in-hospital mortality in NF patients. The lactate/albumin (L/A) ratio has been proposed as a prognostic marker because the ratio is associated with multiple organ failure and mortality in critically patients [17, 18]. However, the prognostic performance of L/A ratio has not yet been reported among patients with necrotizing fasciitis. In this study, we evaluated the prognostic performance of the L/A ratio and lactate levels for predicting outcome in NF.

## 2. Materials and Methods

### 2.1. Patient Selection

The institutional review board of two hospitals approved this study. We followed the method of Shin et al.'s study [19]. The medical records of patients who met the inclusion criteria of surgically proven NF and who received management between 2015 and 2020 in two tertiary hospitals were reviewed. Selected comorbidities and initial laboratory values were extracted through medical chart review. All patients were assessed by emergency physicians as soon as they were admitted. They received broad-spectrum antibiotic treatment for anaerobic and aerobic bacteria as well as early surgical debridement including fasciotomy or primary amputation after diagnosis. Each patient's medical record was screened for documentation of NF to confirm the diagnosis. Blood samples were collected in the ED upon arrival, so the lactate and albumin levels were determined. Variables were compared between survivors and nonsurvivors groups.

### 2.2. Data Collection and Measurement

Age, sex, vital signs in the ED, the presence of comorbidities (i.e., heart disease, pulmonary disease, liver disease, kidney disease, and diabetes), serum lactate, albumin, and SOFA score were analyzed. After data collection was completed, random chart reviews were performed to ensure accuracy. Patients with in-hospital mortality were defined as a death occurring in the hospital after admission, also known as the ‘nonsurvivors group', and otherwise as the ‘survivors group.' We defined these variables as follows: shock status, episodes of systolic blood pressure less than 90 mmHg at the ED; hypothermia, body temperature less than 36°C at the ED; hyperthermia, body temperature ≥ 38°C at the ED; acidosis, pH less than 7.35 in the arterial blood gas test at the ED; coagulopathy, a prolonged prothrombin time test (international normalized ratio) result greater than 1.5 or platelet counts less than 100 × 10^3^ uL at the ED; and hypoxia, episodes of SpO2 < 90%, oxygen saturation less than 90% at the ED. Prediction accuracy of in-hospital mortality was assessed using the area under the receiver operating characteristic (AUROC) curve. Prognostic accuracy was assessed in all patients and in subgroups based on lactate levels. For comparison of AUROC curves of two diagnostic modalities (L/A ratio, lactate), we used a test for dependent receiver operating characteristic curves. The cutoff point was determined using the Liu method that maximizes sensitivity and specificity. To adjust for potential confounders in the association between the L/A ratio and in-hospital mortality, individual factors with a *P* value less than 0.05 were used for multiple logistic regression. Statistical significance was defined by a *P* value <0.05.

## 3. Results

Baseline characteristics of survivors and nonsurvivors groups are listed in Table 1. The overall in-hospital mortality was 7.63% in all the patients. The overall median age of patients was 59.8 (IQR 50.2–75.4), and 163 (62.2%) patients were male. The overall median maximum SOFA score initially was 7 (IQR 5–11). In laboratory tests, the nonsurvivor group showed lower albumin levels (median, 2.6 g/dL vs 3.1 g/dL), higher lactate levels (median, 4.9 mmol/L vs 2.8 mmol/L), and higher L/A ratio (median, 1.8 vs 1.1) than the survival group (*P* < 0.05, all).  Table 2 shows comparisons between AUROC values and the cutoff point of the L/A ratio for in-hospital mortality. The AUROC value of the L/A ratio for predicting mortality (0.76, 95% confidence interval (CI) 0.70–0.82; *P* < 0.01) was higher than that for lactate alone (0.71, 95% CI 0.65–0.77; *P* < 0.01). The AUROC value of the L/A ratio was significantly higher than that of lactate regardless of lactate level (normal: 0.75, 95% CI 0.70–0.80 vs. 0.64, 95% CI 0.59–0.69, *P* < 0.01; intermediate: 0.69, 95% CI 0.63–0.75 vs. 0.59, 95% CI 0.53–0.75, *P* < 0.01; and high: 0.72, 95% CI 0.68–0.76 vs. 0.68, 95% CI 0.64–0.72, *P*=0.015).  Table 3 compared in-hospital mortality according to initial lactate level using the cutoff point for L/A ratio and albumin level. The mortality rates were significantly higher when the L/A ratio was above the cutoff point regardless of lactate level (normal: 6.9% vs. 2.2%, *P* < 0.01; intermediate: 13.0% vs. 3.9%, *P* < 0.01; and high: 15.6% vs. 7.2%, *P* < 0.01). A significant trend in higher mortality was also observed in hypoalbuminemia patients compared with patients with normal albumin. After adjusting for several confounding factors such as shock, acidosis, SOFA score, chronic kidney disease, and diabetes, we found a significant association between the L/A ratio and in-hospital mortality (adjusted odds ratio (OR) 1.48, 95% CI 1.30–1.75; *P* < 0.01; Table 4).

## 4. Discussion

The diagnostic performance of the L/A ratio was better than that of single lactate for predicting in-hospital mortality in NF patients. Improved prognostic accuracy was consistent in subgroups of lactate levels (low, intermediate, and high), the AUROC of the L/A ratio was relatively fair for predicting mortality (AUROC ≥ 0.70). In addition, the L/A ratio cutoff and hypoalbuminemia showed further discriminative value for in-hospital mortality than single lactate even in patients with normal or intermediate lactate levels. Many studies have demonstrated that either admission lactate or peak lactate concentration is associated with mortality in adults. Our results are in line with recently published findings that suggest that blood lactate concentration upon ED arrival is predictive of mortality, independent of SOFA score [10, 20–22]. Some theories exist regarding the reasons why lower serum albumin levels, or hypoalbuminemia, may be associated with poor outcomes. Because synthesis and distribution of albumin may be directly associated with serum albumin level, factors that could affect albumin synthesis, distribution, or both need to be considered. The L/A ratio has been proposed as a marker for combined interpretation of lactate and albumin levels; some studies have investigated its usefulness as a prognostic marker in sepsis or ICU patients [23, 24]. To the best of our knowledge, our study is the first to evaluate the L/A ratio and in-hospital mortality for NF patients.

The L/A ratio has potential benefits for prognostication of NF patients considering the results from our study and previous studies. The two markers independently predict mortality, and a combination of both parameters might increase the predictive value. Furthermore, normal or intermediate levels of lactate might be misinterpreted as a good prognosis. The L/A ratio might be used for additional identification of high-risk patients. In addition, our study suggests that a simple approach might provide further prognostic information using hypoalbuminemia and categories of lactate level.

There were several limitations of this study. First, this study was retrospective in nature and, thus, has inherent limitations with regards to selection bias. The authors who were aware of the potential biases held multiple meetings to ensure patients were correctly identified. Second, the data were collected from two centers limiting the generalizability of these results. The validity of the result still requires other external validation to confirm. Third, we only included patients on whom lactate and albumin were both taken. Finally, we did not have enough information to compare the performance of the L/A ratio with other scoring systems such as APACHE II.

## 5. Conclusions

The prognostic performance of the L/A ratio was better than a single lactate measurement for predicting in-hospital mortality in NF patients. This ratio was a useful prognostic factor regardless of initial lactate level. Aggressive intervention and intensive care were necessary for high-risk NF patients upon ED arrival.

## Figures and Tables

**Table 1:** Comparison of baseline characteristics between survival and nonsurvival groups.

Characteristics	Survivors (*n* = 242)	Nonsurvivors (*n* = 20)	*P* value
Age (years)	58.3 (39.1–62.3)	60.7 (41.4–79.5)	0.89
Sex (male)	150 (62.0%)	13 (65.0%)	0.57

ED vital signs			
Hypoxia	24 (10.0%)	3 (15.0%)	0.07
Shock	49 (20.2%)	6 (30.0%)	<0.01
Hyperthermia	143 (59.1%)	12 (60.0%)	0.91
Hypothermia	43 (17.8%)	3 (15.0%)	0.84
SOFA score	4 (0–6)	9 (5–23)	<0.01

Laboratory test			
Acidosis	45 (18.6%)	6 (30.0%)	0.02
Coagulopathy	39 (16.1%)	4 (20.0%)	0.44
Lactate (mmol/l)	2.8 (0.5–5.6)	4.9 (1.2–9.8)	<0.01
Albumin (g/dl)	3.1 (2.1–4.8)	2.6 (1.9–3.6)	<0.01
Lactate/albumin ratio	1.1 (0.8–1.8)	1.8 (1.1–2.8)	<0.01

Comorbidities			
Heart failure	38 (15.7%)	3 (15.0%)	0.97
Diabetes mellitus	63 (26.0%)	7 (35.0%)	0.04
Liver cirrhosis	48 (19.8%)	4 (20.0%)	0.15
Chronic kidney disease	75 (31.0%)	9 (45.0%)	0.01
Chronic lung disease	29 (12.0%)	2 (10.0%)	0.66
ICU lengths (day)	5.4 (2.1–11.8)	9.2 (1.5–19.7)	<0.01

Values are represented as median (interquartile range). Numbers in parentheses denote percentages. ED, emergency department; ICU, intensive care unit; SOFA, sequential organ failure assessment.

**Table 2:** AUROC and L/A ratio cutoff value for in-hospital mortality.

	AUROC for in-hospital mortality (95% confidence interval)			Lactate/albumin ratio cutoff point		
	Lactate	L/A ratio	*P*	Cutoff value	Sensitivity	Specificity
All patients	0.71 (0.65–0.77)	0.76 (0.70–0.82)	<0.01	1.61	0.75	0.81

Lactate level (mmol/L)						
Normal lactate (<2.0)	0.64 (0.59–0.69)	0.75 (0.70–0.80)	<0.01	0.75	0.76	0.82
Intermediate lactate (2.0 ≦ *x* < 4.0)	0.59 (0.53–0.75)	0.69 (0.63–0.75)	<0.01	1.36	0.63	0.85
High lactate (≧4.0)	0.68 (0.64–0.72)	0.72 (0.68–0.76)	0.015	2.48	0.71	0.83

**Table 3:** In-hospital mortality according to lactate level, lactate/albumin (L/A) ratio, and albumin level.

	In-hospital mortality	*P*
Lactate and L/A ratio		
Normal lactate level		<0.01
L/A ratio < cutoff point (0.75) (*n* = 45)	1 (2.2%)	
L/A ratio ≧ cutoff point (0.75) (*n* = 29)	2 (6.9%)	

Intermediate lactate level		<0.01
L/A ratio < cutoff point (1.36) (*n* = 51)	2 (3.9%)	
L/A ratio ≧ cutoff point (1.36) (*n* = 23)	3 (13.0%)	

High lactate level		<0.01
L/A ratio < cutoff point (2.48) (*n* = 69)	5 (7.2%)	
L/A ratio ≧ cutoff point (2.48) (*n* = 45)	7 (15.6%)	

Lactate and albumin level		
Normal lactate level		<0.01
Albumin < 3.0 (*n* = 33)	2 (6.1%)	
Albumin ≧ 3.0 (*n* = 41)	1 (2.4%)	

Intermediate lactate level		<0.01
Albumin < 3.0 (*n* = 39)	4 (10.3%)	
Albumin ≧ 3.0 (*n* = 35)	1 (2.9%)	

High lactate level		<0.01
Albumin < 3.0 (*n* = 60)	8 (13.3%)	
Albumin ≧ 3.0 (*n* = 54)	4 (7.4%)	

**Table 4:** Multivariable logistic regression.

	Odds ratio	95% CI	*P*
Shock	1.02	1.00–1.04	0.19
Acidosis	0.98	0.82–1.14	0.58
Chronic kidney disease	1.35	1.29–1.41	0.02
Diabetes mellitus	1.74	1.25–2.16	0.26
SOFA score	1.21	1.14–1.19	0.01
Lactate/albumin ratio	1.48	1.30–1.75	<0.01

## Data Availability

Data are available on request.

## Abbreviations

AUROC: Area under the receiver operating characteristic curve
CI: Confidence interval
ED: Emergency department
NF: Necrotizing fasciitis
OR: Odds ratio
SOFA: Sequential organ failure assessment.
